# Prescriptions

**Published:** 1897-06

**Authors:** 


A PAINLESS VESICANT.

℞. Menthol and chloral hydrate, of each.................1 part
Cacao butter.............2 parts
Spermaceti.............4 parts
M. Sig: Mix to form a paste.

When spread on a cloth or upon adhesive plaster, and applied to the surface of the skin, this paste acts similarly to cantharides plaster.—*Rev. Medico-Pharm.*

ECZEMA.

In the obstinate inveterate forms of eczema, Lassar extols the application of Wilkinson's ointment, originally introduced as a remedy for scabies. It is composed as follows:

℞. Sulphur,......................
Ol rusci. .. ...........aa 4 parts
Cretæ preparatæ... .....1 part
Sapon moll,...................
Paraffin moll.........aa 8 parts
—*British Journal Derm.*, viii., 155.

A LAXATIVE POWDER FOR CONSTIPATION IN CHILDREN.

℞. Bicarbonate of sodium.....3 dr.
Powdered rhubarb.........2 ozs.
Sulphate of sodium.........1 oz.
Oil of peppermint.....20 drops.
M. Sig: Half to one teaspoonful of this powder may be given in the morning before breakfast.—*Journal de Medecine de Paris.*

